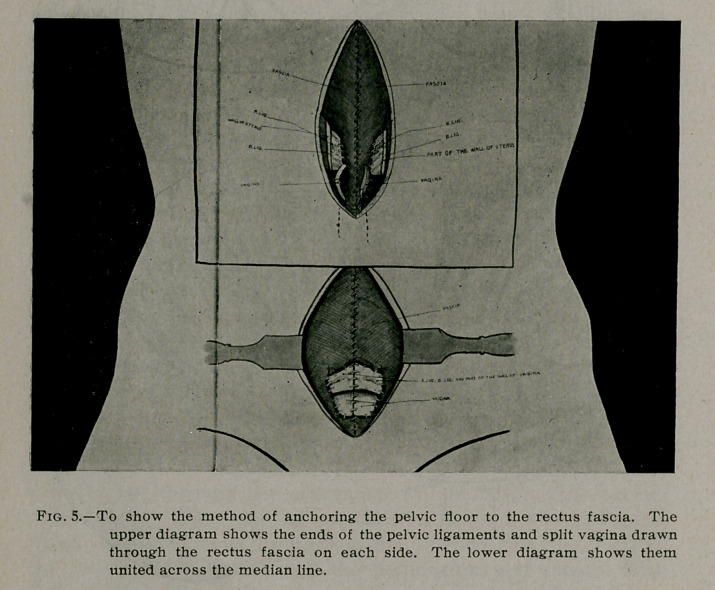# A New Operation for Hernia of the Pelvic Floor (Procidentia) with Report of a Case1Read at the meeting of the Clinical and Pathological Section of the Academy of Medicine of Cleveland, March 3, 1905.

**Published:** 1905-09

**Authors:** George W. Crile

**Affiliations:** Cleveland, Ohio, 216 The Osborne


### A New Operation for Hernia of the Pelvic Floor (Procidentia) with Report of a Case.[1]

BY GEORGE W. CRILE, M. D., Cleveland, Ohio.

[From *The Cleveland Medical Journal*, July, 1905.]

*Synopsis.*—Mrs. K. Consultation: Dr. H. L. Hall, North Amherst. Complete hernia of the pelvic floor. First operation anterior and posterior plastic with repair of perineum. Recurrence in four months. Second operation vaginal hysterectomy with fixation of the round and broad ligaments to the fornix. Recurrence after six months. Third operation, laparatomy with suspension of the pelvic ligaments and vagina to the abdominal wall. Three years and one month later no recurrence.

*History.*—The patient had the diseases of childhood; she was married at 20, and had nine children, the youngest of whom is now 16 years old. She has never been well since her first child was born. Her menstrual periods have been regular, and she has had no disease bearing upon the present ailment. She complains of pain and dragging sensations in both sides and in the pelvis, of severe backache, greatly increased on walking. The bowels have been constipated, and there has been great difficulty in micturition. There is a constant vaginal discharge, with loss of appetite, spirits and strength. She also complained of the soreness incident to the ulcer on the cervix.

*Physical Examination.*—The patient is well-built, fairly well nourished, short and stout, with a very large accumulation of adipose in the abdomen. No edema. Circulation and respiration are normal with the exception of slight arterial sclerosis. On standing the uterus, the vagina, the broad and round ligaments, the rectum, the bladder, and a considerable portion of the small intestine form a large oval-shaped protrusion, extending almost to the knees. On the cervix there is extensive ulceration. The mucous membrane is greatly thickened and hardened and is the seat of chronic inflammation.

*The first operation* consisted of an amputation of the cervix, and a reduction of the hernia with extensive anterior and posterior plastic operations upon the vaginal wall, together with as extensive a repair of the perineum as was possible. The difficulty in doing this portion of the operation was due to the almost complete disappearance of the rectovaginal septum, with marked stretching of all the parts in the extensive descent of the hernia.

---

1. Read at the meeting of the Clinical and Pathological Section of the Academy of Medicine of Cleveland, March 3, 1905.

Fig. 1.—Schematic representation of the pelvic contents in their normal relations. (Frontal section.)

Fig. 2.—Showing relations of pelvic structures in pelvic hernia.

FIG. 3.—Showing pelvic hernia reduced, hysterectomy partially completed, vagina split. The free end of the broad ligament and the corresponding half of the split vagina is to be drawn up through the rectus fascia on each side.

The patient had had a chronic cough which added greatly to the stress upon the operation.

After four months recurrence was quite marked, though not complete.

*Second Operation.*—At this operation a vaginal panhysterectomy was done and the round and broad ligaments were sutured to the vaginal wall, closing the top of the pelvis and strengthening the floor as much as available material would permit.

Recurrence of the hernia again took place after six months. At this time the floor of the pelvis and the rectovaginal septum had entirely given away, and a symmetrical pouch-like sac, containing a very considerable portion of the large and small intestines, was suspended between the thighs. Her sufferings were as great as before and both operations had given her no measure of relief. The Pryor operation, that of obliterating the vagina, was then contemplated, but not accepted by the patient.

*Third Operation.*—After considering a number of plans both theoretically and upon the cadaver, the following was executed: in the Trendelenburg posture a median incision of good length was made, approximately one-fourth of the entire abdominal contents were withdrawn from the hernial sac, the pelvic floor studied, and the hernia reduced. The bladder was found well down in this cavity and totally prolapsed. An anterio-posterior incision was made across the middle of the floor of the pelvis, dividing the vagina into two lateral halves. The vaginal mucous membrane of the part to be brought through the abdominal wall was removed. The bladder was separated from the vagina for some distance downward. It was found that the vagina and the floor of the pelvis had been so stretched that they could be easily brought out through the abdominal wound beyond the surface of the skin. After making an incision through the abdominal fascia, four cm. from the median line on each side, the fibers of the recti were separated and the peritoneum perforated. Each half of the split vagina with the attached utero-sacral and utero-pelvic ligaments, and all the other structures of the floor of the pelvis together with the round and the broad ligaments, were drawn out through these openings on each side of the median incision. While held well up in place so that the top of the incised vagina presented closely against the under surface of the peritoneum, the peritoneum was closed around this portion with plain catgut. The original peritoneal incision, the muscle and the external fascia were then closed, the latter by continuous sutures of chromicised gut, after which the freed ends of the vagina and pelvic floor, which had been drawn up through the lateral openings in the peritoneum, recti and fascia, were united in the middle line by means of chromicised gut. The skin was then closed.

FIG. 4.—Sagital section showing completed operation.

The patient made a good recovery from the operation and was discharged from the hospital in three and one-half weeks.

*Clinical Results.*—For some time after the operation the patient felt a sensation of dragging upon the wound and experienced some pain. This passed away after several months. She has been doing her usual work, and at the present time, more than three years after the operation, there has been no recurrence of the hernia. I have personally examined her at intervals since the operation and have found that the line of apposition has held. During this time she has had a chronic cough in winter, and has been actively engaged in her ordinary domestic duties.

*Comments.*—The difficulties in this operation are due mainly to the great stress upon the pelvic floor in every form of increased intraäbdominal pressure, as coughing, sneezing, laughing, strain-

FIG. 5.—To show the method of anchoring the pelvic floor to the rectus fascia. The upper diagram shows the ends of the pelvic ligaments and split vagina drawn through the rectus fascia on each side. The lower diagram shows them united across the median line.

ing, lifting, and the like. When the natural pelvic floor has once proven itself too weak to take this strain it is manifestly difficult to add sufficient intrinsic strength by any material available in the immediate territory. That this is a practical difficulty is indicated by the 30.2 per cent. of relapses in Hegar's large series, 22 per cent. in Herff's, 22 per cent. in Schmidt's, 20 per cent. in Schultz's, and the like.

The indication for this operation exists only in the cases of complete hernia (procidentia). Indeed it would be quite impossible in the minor degrees of prolapse to carry out this technic for want of sufficient length of ligaments and of vagina to reach to the external fascia. That is to say, the operation is self-limited to proper cases.

My records show 20 operative cases of pelvic hernia, upon which 24 major operations were performed, 16 according to prevailing methods with 25 per cent. recurrences, and eight by the method herein described with no recurrences as yet. There was no mortality by any of the methods.

In conclusion the writer wishes to express his acknowledgement and great appreciation to Dr. C. D. Selby for the drawings which accompany this article.

216 The Osborne.

## Figures and Tables

**Fig. 1. f1:**
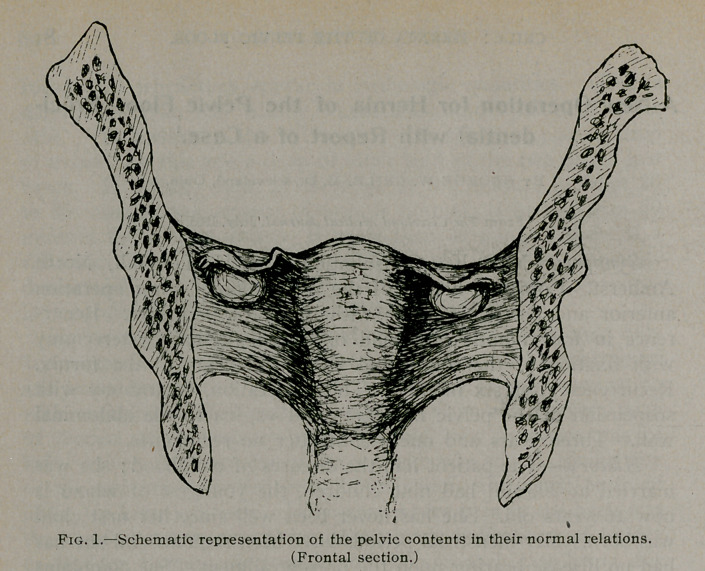


**Fig. 2. f2:**
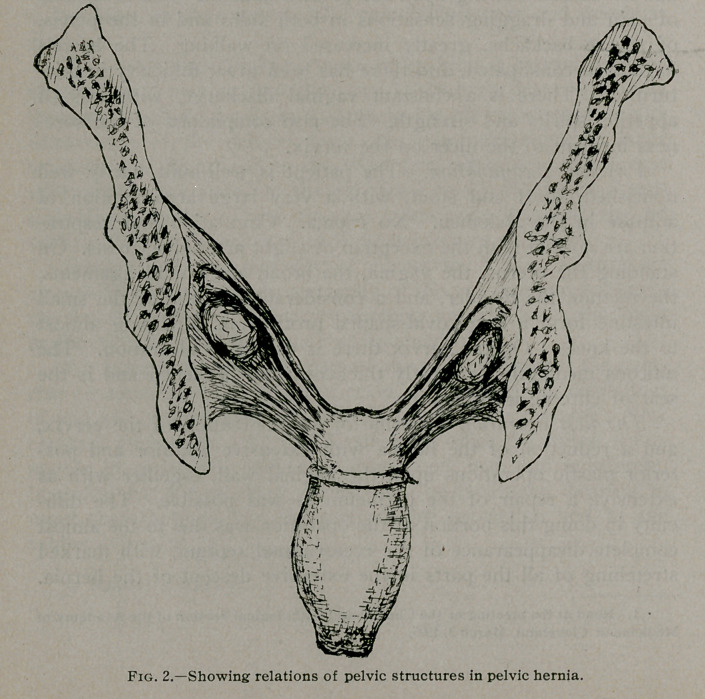


**Fig. 3. f3:**
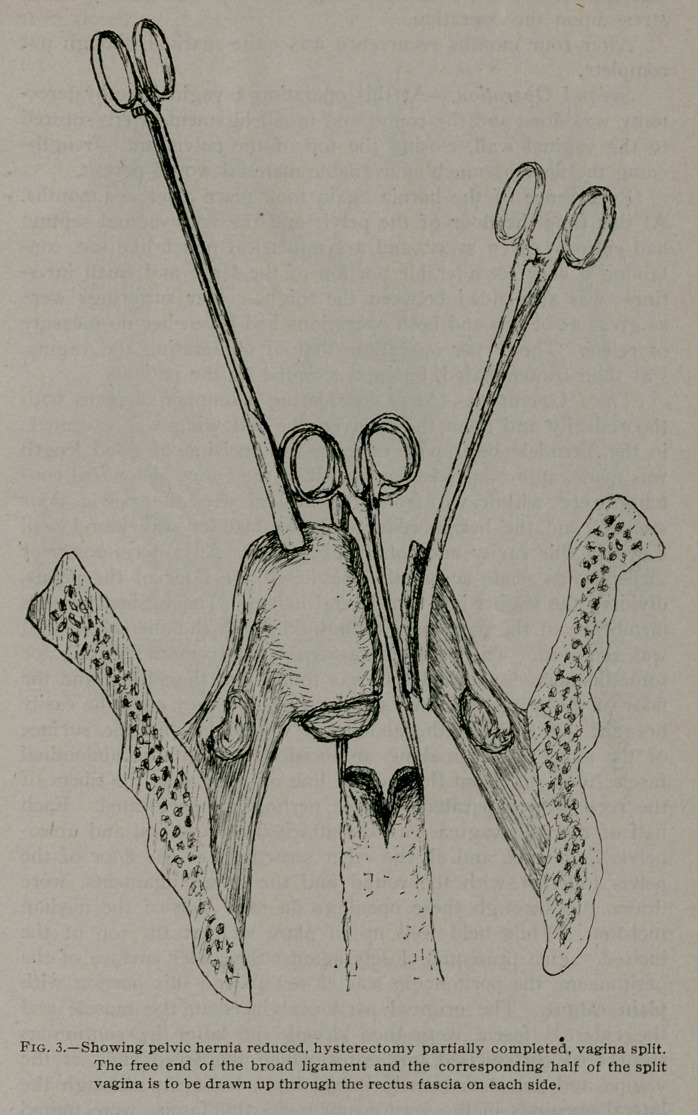


**Fig. 4. f4:**
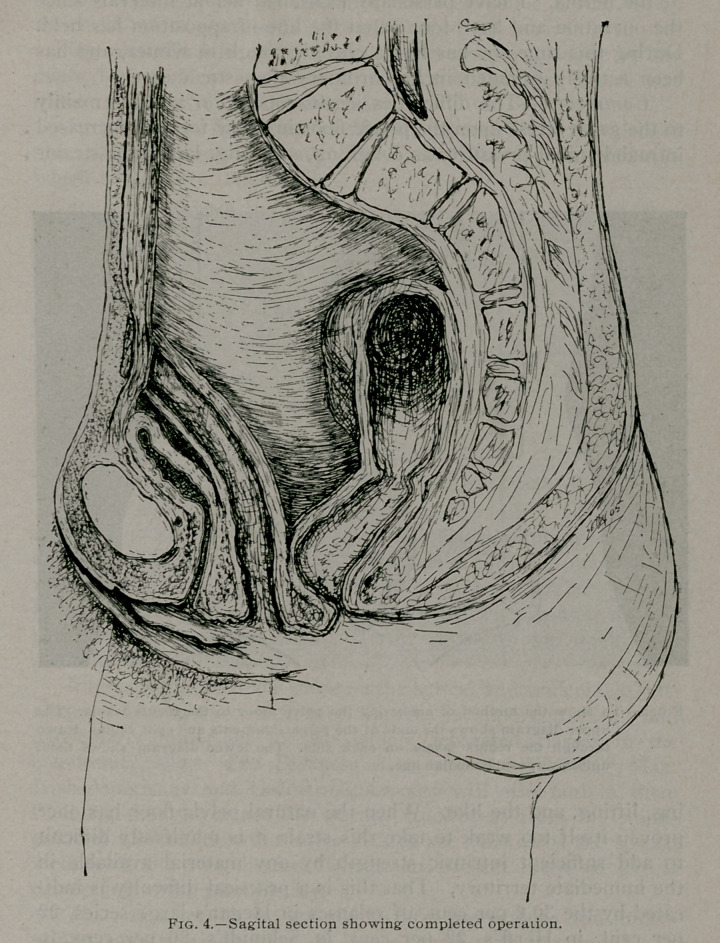


**Fig. 5. f5:**